# Ancient DNA indicates a century of overhunting did not reduce genetic diversity in Pacific Walruses (*Odobenus rosmarus divergens*)

**DOI:** 10.1038/s41598-024-57414-2

**Published:** 2024-04-08

**Authors:** Kendall K. Mills, Kyndall P. B. Hildebrandt, Kathryn M. Everson, Lara Horstmann, Nicole Misarti, Link E. Olson

**Affiliations:** 1 Department of Mammalogy, University of Alaska Museum, 1962 Yukon Drive, Fairbanks, AK 99775 USA; 2 Department of Biology and Wildlife, University of Alaska Fairbanks, Fairbanks, AK 99775 USA; 3 Department of Integrative Biology, Oregon State University, 2701 SW Campus Way, Corvallis, OR 97331 USA; 4 College of Fisheries and Ocean Sciences, University of Alaska Fairbanks, Fairbanks, AK 99775 USA; 5 Water and Environmental Research Center, University of Alaska Fairbanks, Fairbanks, AK 99775 USA

**Keywords:** Evolutionary biology, Population genetics

## Abstract

Pacific Walruses (*Odobenus rosmarus divergens* [Illiger 1815]) are gregarious marine mammals considered to be sentinels of the Arctic because of their dependence on sea ice for feeding, molting, and parturition. Like many other marine mammal species, their population sizes were decimated by historical overhunting in the nineteenth and twentieth centuries. Although they have since been protected from nearly all commercial hunting pressure, they now face rapidly accelerating habitat loss as global warming reduces the extent of summer sea ice in the Arctic. To investigate how genetic variation was impacted by overhunting, we obtained mitochondrial DNA sequences from historic Pacific Walrus samples in Alaska that predate the period of overhunting, as well as from extant populations. We found that genetic variation was unchanged over this period, suggesting Pacific Walruses are resilient to genetic attrition in response to reduced population size, and that this may be related to their high vagility and lack of population structure. Although Pacific Walruses will almost certainly continue to decline in number as the planet warms and summer sea ice is further reduced, they may be less susceptible to the ratcheting effects of inbreeding that typically accompany shrinking populations.

## Introduction

The extinction vortex describes the mutually reinforcing effects of demographic stochasticity, lost genetic variation, and shrinking effective population size that accelerate decline after the number of individuals in a population or species falls below a critical threshold^1^. In theory, populations that have entered the vortex are highly unlikely to escape it. Vortex-like dynamics have been documented in several wild vertebrate populations that have been tracked to extinction^2^, signifying the importance of this phenomenon to the current biodiversity crisis. From the 18th to 20th centuries, commercial overhunting of several marine mammals—driven primarily by demand for fur and blubber—substantially reduced population sizes in dozens of species and presumably drove many near or into the extinction vortex^3–5^. However, the majority of species overhunted in this period rebounded rapidly after industry regulations were implemented and populations were reduced such that hunting was no longer profitable^6–9^. Genetic analyses further indicate that, in many cases, the overhunting that took place over the last two to three centuries had little impact on standing genetic variation^7,10,11^. Studies of marine mammal species that recovered from bottlenecks in this period have provided a more nuanced understanding of how bottlenecks impact genetic diversity and the trajectory of subsequent recovery.

Here we examine how 150 years of commercial exploitation impacted genetic variation in the Pacific Walrus (*Odobenus rosmarus divergens*), a gregarious pinniped hunted extensively for its hide, oil, and ivory tusks^12^. Intensive hunting of this species began in 1867 following the U.S. acquisition of what is now Alaska from Russia, which gave American whalers access to the Bering and Chukchi seas, where they began to hunt walruses in addition to whales^13,14^. In the short period between 1867 and 1887, the Pacific Walrus population was estimated to have been reduced from about 200,000 individuals to 50,000–100,000 individuals^15–17^. It is believed that one-third of Indigenous people in the Bering Strait region died of starvation from 1878–1879 due to diminished walrus harvests^5^. Commercial hunting subsided in 1880 because walruses had already become too scarce to be hunted profitably, but population recovery was so rapid that it attracted a second wave of intensive overhunting only two decades later that peaked around 1920. Numerical catch estimates were not recorded during this period, but historical accounts attest to noticeable depletion of walrus stocks and consequent hardships for Indigenous people^5^. Following a second population recovery, yet another wave of renewed commercial hunting efforts commenced, this one estimated to have once again reduced the population by half^17–19^. Coordinated efforts between Alaska and the U.S.S.R to regulate catches greatly eased hunting pressure beginning around 1960, and by 1980 Pacific Walruses had expanded to occupy all of their previous range and were believed to have recovered to pre-exploitation levels^20^.

The three population contractions of the Pacific Walrus that are reported to have occurred in this period are somewhat unique among pinnipeds, as most were hunted intensely over one contracted period^4^. The duration of a bottleneck is positively correlated with loss of genetic variation^21,22^, and that many marine mammals exhibit high variation despite being overexploited has been attributed to the long lifespan in these species combined with the relatively short period of commercial harvesting, meaning most bottleneck events would have spanned only a small number of generations^17^. It is not obvious if Pacific Walruses can be considered to have undergone three bottlenecks in succession or one protracted event, not only because census data are not always accurate estimates of effective population size, but also because the size at which any given population can be considered to be bottlenecked is dependent upon many demographic and life history characteristics^1,21^. Regardless, both scenarios are expected to have greatly eroded genetic variation. This was the prediction of Sonsthagen et al.^23^, who sequenced 11 microsatellite loci and 350 bp of mitochondrial control region in modern Pacific Walruses and found, instead, that nucleotide and haplotype diversity were high relative to other pinnipeds.

There are three discernible trends among marine mammal species that survived commercial exploitation in the 18th to 20th centuries: rapid population recovery with little loss of genetic variation, as has been reported in New Zealand Fur Seals (*Arctocephalus forsteri*)^9^, Antarctic Fur Seals (*Arctocephalus gazella*)^11^, Humpback Whales (*Megaptera novaeangilae*)^8^, Bowhead Whales (*Balaena mysticetus*)^10^ and Sei Whales (*Balaenoptera borealis*)^24^; rapid population recovery despite significant loss of genetic variation, such as in Narwhals (*Monodon monocerus*)^25^; and a failure to recover to pre-exploitation levels coupled with significant loss of genetic variation, such as in Southern Elephant Seals (*Mirounga leonina*)^26^, Sea Otters (*Enhydria lutris)*^27^, and Hawaiian Monk Seals (*Monachus schauinslandi*)^28^. Current population estimates and the genetic analysis of modern samples by Sonsthagen et al.^23^ suggest Pacific Walruses fall into the first category. To more accurately assess the impact of overhunting on genetic variation in Pacific Walruses, we sequenced 196 bp of mitochondrial D-loop in 28 archaeological samples that dated from 200 to 3300 BP. Several thousand years of presumed low-level subsistence harvests in Alaska and Russia have provided a rich source of archaeological Pacific Walrus specimens that predate the period of commercial overhunting and thus offer a glimpse into a time when the species was relatively undisturbed by human activity. Ancient DNA (aDNA) is highly useful for tracking genetic variation over deep time scales, and thus is often used to measure the lasting impact of specific historical events. For example, analysis of aDNA recovered from bones of ancient Crested Penguins (*Eudyptes* spp.) in New Zealand suggests that, despite considerable range contraction due to centuries of human encroachment, genetic diversity has remained relatively stable^29^. This study also uncovered a now-extinct coastal clade of *Eudyptes*, which attests not only to the ability of aDNA to discover genetic lineages unrepresented in extant populations, but also to the capacity of a well-connected core population to maintain genetic diversity despite local extirpations. Similarly, an analysis of aDNA from Gray Whale (*Eschrichtius robustus*) remains in the Pacific Northwest indicated the effective population size was 3–5 times greater prior to widespread commercial whaling in the nineteenth century, but genetic diversity measurements were not significantly different between ancient and modern samples^30^. In contrast, in a study of the Grauer Gorilla (*Gorilla beringei graueri*), a subspecies of the Eastern Gorilla, aDNA found that habitat fragmentation and poaching coincided with a significant loss of genetic diversity over the last century, largely due to loss of peripheral populations^31^.

Recovery following a bottleneck is impacted by population substructure both before and after the bottleneck event^32,33^, so we investigated a number of longstanding population-level and phylogenetic questions that can be addressed with new DNA sequence data. Two to three subspecies of walrus (*Odobenus rosmarus* [Linnaeus 1758]) are variably recognized: the Atlantic Walrus (*O. rosmarus rosmarus*) is found near Canada, Greenland, and Norway; the Laptev Walrus (*O*. *rosmarus laptevi* [Chapskii 1940]) is restricted to the Laptev Sea of northern Russia; and the Pacific Walrus inhabits the Chukchi and Bering Seas and is by far the most numerous of the three subspecies^12^. The extent of population structure present within Pacific Walruses is unclear; previous studies found evidence of only mild structure despite their expansive range and suggested this subspecies is more panmictic than Atlantic Walruses^20,34,35^. The taxonomic status of the Laptev Walrus is also debated, as previous analyses have found them to be genetically and morphologically indistinguishable from Pacific Walruses^35^. However, the two putative subspecies are separated by over 500 km, and no interbreeding is known to occur between them^20,35^; the Laptev Walrus thus may still be an important population segment for conservation purposes. Finally, the biogeographic history of the species as a whole is enigmatic. It is unknown where *O. rosmarus* originated, when or by what route it spread to the other side of North America, or how long the three putative subspecies have been genetically isolated from each other. To address these questions, we supplemented our mitochondrial dataset with previously published sequences from Atlantic and Laptev Walruses^23,35^.

Although Pacific Walruses are thought to have recovered to pre-exploitation levels, they now face an altogether different threat in the form of climate change. Walruses are highly dependent on floating sea ice as haul-outs during feeding, pupping, and molting, and for this reason, they are thought to be especially vulnerable to the effects of global warming^36,37^. The spatial extent of summer sea ice in the Arctic Ocean has declined dramatically in the last decade^38,39^; consequently, walruses have been observed using coastal haul-outs, which not only decreases the amount of prey within their feeding range, but has also led to mass mortality events caused by trampling^40^. For example, after a single haul-out event in 2009 in Icy Cape, Alaska, 131 individuals were found dead on the beach, the majority of which were calves that died by asphyxiation due to having been trampled^41^. The continued loss of suitable Pacific Walrus habitat is expected to reduce carrying capacity in the coming decades, and comparing DNA sequences before and after the period of commercial overhunting will allow us to better determine how previous harvest levels impacted genetic variation, whether the species now faces the threat of climate change with less variation than they maintained historically, and what degree of population loss can be tolerated before genetic variation begins to decline. Falling Pacific Walrus numbers may have far-reaching effects on the Arctic ecosystem, as their foraging behavior provides essential nutrient mixing and because they are thought to prey on other pinnipeds when unable to access benthic prey^40,42^. And unlike the threat Pacific Walruses faced from overhunting, from which they recovered by recolonizing their historic range and rebounding in terms of population size, climate change poses a fundamentally different problem in that suitable habitat will be permanently lost. To this end, we also modeled effective population size over deeper timescales to infer how Pacific Walrus numbers responded to climatic changes in the past.

## Methods

### Specimen collection

**Figure 1:**
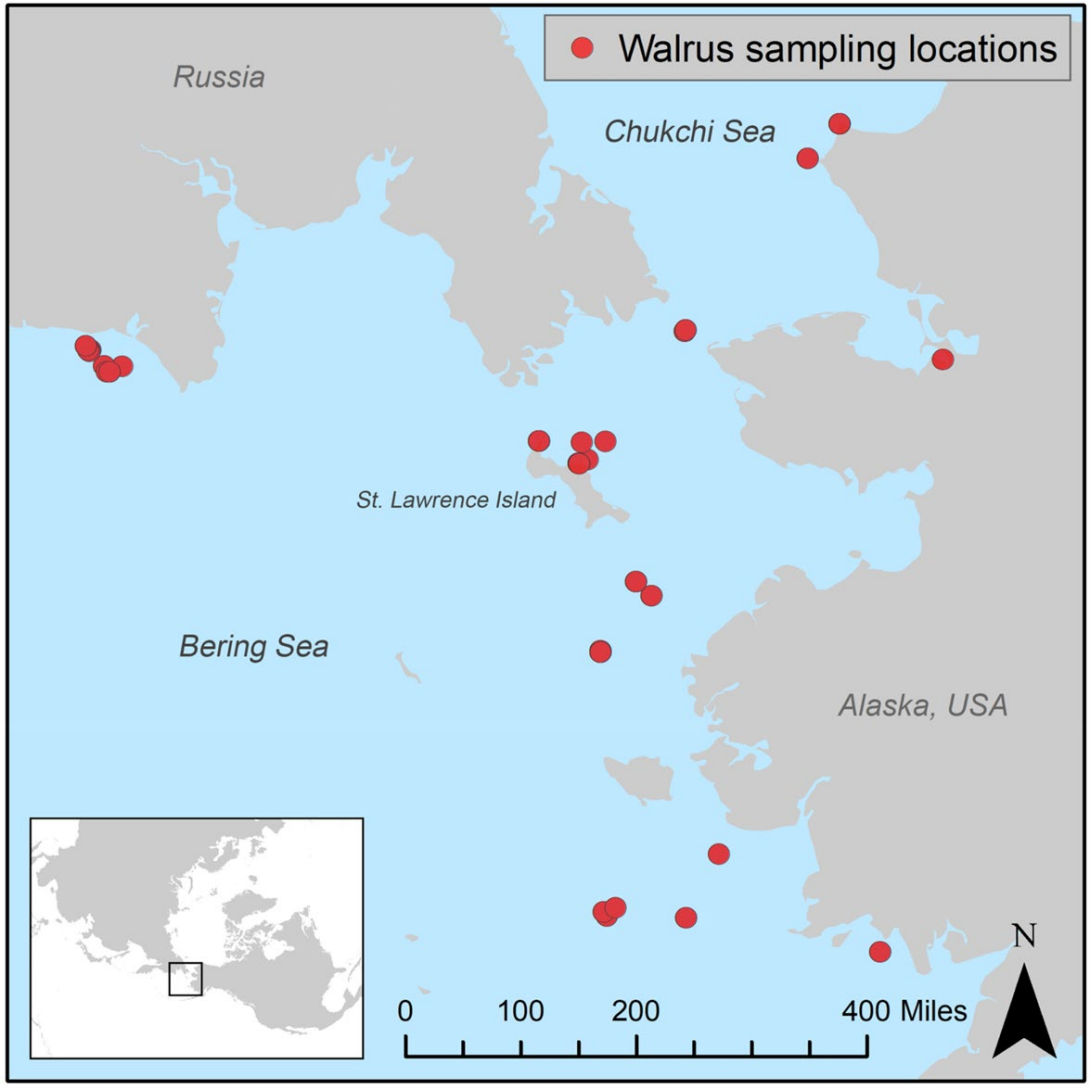
Locations of modern samples used in this study. Modern Pacific Walrus samples were obtained from the 2014–2016 subsistence harvests in Gambell and Savoonga on St. Lawrence Island, Alaska, and the remaining samples from 1933 to 2015 were provided by the North Slope Borough Department of Wildlife Management in Utqiaġvik, Alaska; the University of Alaska Museum (UAM); and the U.S. National Museum of Natural History, Smithsonian Institution (USNM).

Modern Pacific Walrus samples (n = 13) were obtained from the 2014–2016 subsistence harvests in Gambell and Savoonga on St. Lawrence Island, Alaska, through agreements with the Alaska Native subsistence hunters, the Eskimo Walrus Commission, the Alaska Department of Fish and Game, and the U.S. Fish and Wildlife Service (Fig. 1). Additional samples (n = 75) from 1933 to 2015 were provided by the North Slope Borough Department of Wildlife Management in Utqiaġvik, Alaska; the University of Alaska Museum’s (UAM) Mammal Collection; and the U.S. National Museum of Natural History, Smithsonian Institution (USNM). Archaeological specimens from excavations along the Bering and Chukchi seas and from the southern coast of the Alaska Peninsula were obtained from UAM’s Archaeology Collection and from UIC Science, LLC in Utqiaġvik, Alaska (n = 26). A more detailed account of the collection efforts is available in^43^. A list of all specimens included in this study and their associated data can be found in Table S1.

### Specimen dating

Archaeological samples (n = 26) were dated using accelerator mass spectrometry (AMS) at the Center for Applied Isotope Studies at the University of Georgia, Athens, Georgia, USA, or at the Scottish Universities Environmental Research Center (SUERC), Glasgow, Scotland, UK. Radiocarbon dates were calibrated using OxCal 4.3 with the IntCal13 and Marine13 calibration curves^44^. Sample selection focused on samples that could be securely dated. The selected walrus samples were (1) clearly associated with terrestrial materials, either terrestrial mammal bone or short-lived plants, or (2) from a context that had been or could be securely dated. In all cases, we preferentially selected dense materials that were less likely to have been contaminated by marine mammal oils or other substances. Human artifacts were avoided because there have been instances of very ancient materials being used in their manufacture (e.g.,^45^).

### Amplification of archaeological and historic samples

Archaeological and historic bone samples were obtained from material archived at the University of Alaska Museum and the U.S. National Museum of Natural History, Smithsonian Institution (Table S1). Bone was subsampled using a Dremel tool equipped with a cutting disk or drill bit. The cutting disk and drill bit were sterilized in a dilute solution of bleach, rinsed in ethanol, and flame sterilized between each sample. The bone surface was cleaned with a dilute solution of bleach prior to sampling, or, for smaller fragments, submersed in bleach followed by a soak in sterile distilled deionized water to remove any surface contaminants^46^. If sampled using the drill bit, the resulting powder was used for DNA extraction. If the sample was cut or was itself a bone fragment, the bone was subsequently powderized using a Freezer/Mill^®^ High Capacity Cryogenic Grinder (Spex Sample Prep).

DNA was extracted from 200 to 500 mg of powderized bone using the silica-based protocol of Rohland et al.^47^ and eluted in 50 mL TE with 1.5 mL Tween20. Extractions were performed in a dedicated aDNA facility at UAM, which is housed in a PCR-free building. Prior to this project, no walrus material had been extracted in this laboratory. Standard ancient DNA protocols were strictly followed^48^.

Due to the fragmented nature of historic and aDNA samples, short overlapping fragments of the hypervariable mitochondrial D-loop were amplified using two pairs of previously published primers^33^. The greatest polymorphism was observed in the 208-bp region amplified using primer pair DLF2 and DLR2, which was therefore prioritized. Amplifications consisted of 1U/rxn Invitrogen Platinum Taq DNA Polymerase High Fidelity, 1X High Fidelity PCR buffer, 2.0 mM MgSO_4_, 0.2 mM dNTP, 0.5 µM each primer, and 1 uL unquantified, undiluted DNA extraction in 15 uL reactions over 60 cycles, with an annealing temperature of 52 °C.

### Amplication of modern samples

Genomic DNA from modern samples (Table S1) was extracted from frozen tissue using the ethanol precipitate animal tissue protocol from the PureGene kit (Gentra Systems Inc., Minneapolis, Minnesota, USA). Primers DLF1 and DLR2 from^33^ were used to amplify a longer portion of the mitochondrial D-loop than was recovered for ancient samples. Amplifications were performed using 1.25U Promega GoTaq, 5X GoTaq reaction buffer (1.5 mM MgCl_2_), 0.2 mM dNTP, 0.3 µM each primer (DLF1 and DLR2), and 0.5 uL 1/10 diluted, unquantified extraction in 25 uL reactions over 40 cycles, with an annealing temperature of 54 °C.

### Sequencing and sequence analysis

PCR products were purified using ExoSAP-IT (USB Corp., Cleveland, Ohio, USA) and sequenced for both strands at Keck Biotechnology Resource (New Haven, Connecticut, USA). In addition, 13 Pacific, 27 Atlantic, and five Laptev Walrus sequences from^33^ were retrieved from GenBank (EU728456–EU728487), as were mitogenomes for the Northern Fur Seal (*Callorhinusursinus*; MG916809), Steller Sea Lion (*(Eumetopias jubatus*; GU475464), and California Sea Lion (*Zalophus californianus*; CM019821).

Sequences were aligned in Geneious v.2022.1 using Clustal Omega^49^. A repetitive region of approximately 12 bp was masked due to its susceptibility to replication slippage during PCR. Because only a short region was recovered for most aDNA samples, subsequent analyses made use of two separate alignments: a longer alignment that included only modern samples (644 bp) and a shorter alignment that included modern and aDNA samples (196 bp).

To examine the genetic variation present among the nominal subspecies and between modern and ancient Pacific Walruses, nucleotide and haplotype diversities were calculated for each sample set in R using PEGAS^49^, and Tajima’s D and Fu’s *F*_*s*_ were calculated in DnaSP 6^50^. Median-joining haplotype networks were constructed for both alignments using the PEGAS function in HaploNet to illustrate relationships among the three subspecies and the distribution of ancient haplotypes among extant haplotypes. In addition, we estimated a Bayesian phylogenetic tree in BEAST2.0^51^ based on the short alignment. A best-fitting model for the unpartitioned data was found using bModelTest^52^.

To estimate demographic histories, an Extended Bayesian Skyline Plot was constructed in BEAST 2.0^51,53^ using the long alignment (modern sequences only; Table S1). The same analysis was repeated for the modern Atlantic sequences from^33^. The BEAST package bModelTest was used to identify the best evolutionary model, which was HKY + I + G for both subspecies^52^. A substitution rate of 7.5 × 10^−8^ substitutions/site/year with a strict molecular clock model and a generation time of 10 years^17^ were used to unscale the change in effective population size over time. This substitution rate was estimated previously in Southern Elephant Seals (*Mirounga leonina*), a related pinniped^28^. The MCMC sample was run for 50,000,000 iterations with genealogies sampled every 5000 iterations and 10% discarded as burn-in. Convergence and effective sample sizes (ESS) were examined using Tracer 1.7.1^54^.

## Results

We generated new sequences for 88 modern and 26 archaeological Pacific Walrus individuals (Table S1). All sequences and alignments were deposited in GenBank (OQ174727–OQ174840). Of the 26 ancient samples, 15 could be confidently dated using the methods described above. All 15 of these predated the period of intensive walrus hunting in the nineteenth century (Table 1). After adding sequences from^37^, the long alignment consisted of 644 bp from 91 Pacific, 27 Atlantic, and 5 Laptev Walruses, and the short alignment consisted of 196 bp from 26 archaeological Pacific, 101 modern Pacific, 27 Atlantic, and 5 Laptev Walruses.

**Table 1:** Localities and estimated ages of 15 archaeological samples used for aDNA analysis. Sequence data were collected from an additional 11 archaeological samples whose age could not be accurately estimated.

Sample	Age (BP)	Locality
UAM:Arc:UA2012-052-2564	130–230	E Chukchi (Nuvuk)
UAM:Arc:UA68-086-0066	280–340	St Lawrence Island
UAM:Arc:UA68-086-0004	280–340	St Lawrence Island
UAM:Arc:UA75-009-XPH-00001B	320–420	Point Hope
SL2-HMFLI	360–500	Point Franklin
SL2-3178	360–500	Point Franklin
SL1-855	360–580	Point Franklin
SL1-676	380–570	Point Franklin
UAM:Arc:UA75-009-XPH-00001A	490–570	Point Hope
UAM:Arc:UA2012-051-5338	850–980	E Chukchi (Birnirk)
UAM:Arc:XPM-00001-3413	2780	SE Bering (Hot Springs)
UAM:Arc:XPM-00001-3469	2780	SE Bering (Hot Springs)
UAM:Arc:XPM-00001-6790	2500–3500	SE Bering (Hot Springs)
UAM:Arc:XPM-00001-27006	2500–3500	SE Bering (Hot Springs)
UAM:Arc:XPM-00001-40221	2500–3500	SE Bering (Hot Springs)

Both nucleotide and haplotype diversity were significantly higher in Pacific than in Atlantic Walruses, which is consistent with the former having a much larger effective population size^36,57^. Genetic diversity appears to have declined only slightly between the ancient and modern samples, which was unexpected given the presumed intervening bottleneck due to overhunting. Significantly negative Tajima’s D and Fu’s *F*_*s*_ values for modern samples suggest recent population expansion among Pacific Walruses and, possibly, to a lesser extent in Atlantic Walruses (Table 2).

**Table 2:** Estimates of genetic diversity and demographic history for all subspecies.

Subspecies	Nucleotide diversity (π)	Haplotype diversity (*h*)	Tajima's D	Fu’s *F*_*s*_
Short alignment (196 bp)				
*O. divergens*	0.0252 (0.0225–0.0279)	0.9867 (0.986–0.988)	− 0.9191	− **84.7090**
*O. divergens* (arch.)	0.0297 (0.0238–0.0357)	0.9901 (0.987–0.993)	− 1.2407	− **20.4886**
*O. laptevi*	0.0052 (0.0009–0.0095)	0.9000 (0.783–1.02)	0.2431	− 0.4750
*O. rosmarus*	0.0064 (0.0046–0.0082)	0.7208 (0.694–0.747)	− 0.6243	− 2.2104

Long alignment (644 bp)				
*O. divergens*	0.0096 (0.0086–0.0106)	0.9900 (0.989–0.991)	**− 1.4831**	**− 33.7924**
*O. laptevi*	0.0025 (0.0007–0.0043)	0.8000 (0.687–0.913)	0.6990	0.2760
*O. rosmarus*	0.0062 (0.0049–0.0075)	0.9231 (0.909–0.937)	0.0606	**− 5.4541**

The short alignment includes consists of 26 archaeological samples and 123 modern samples, while the long alignment consists of only the 123 modern samples. Estimates were calculated for each sample set in R using PEGAS^51^. Significant values are in boldface (for Tajima’s D, *p* < 0.10; for Fu’s *Fs*, *p* < 0.02). 95% confidence intervals for *π* and *h* are given in parentheses.

**Figure 2:**
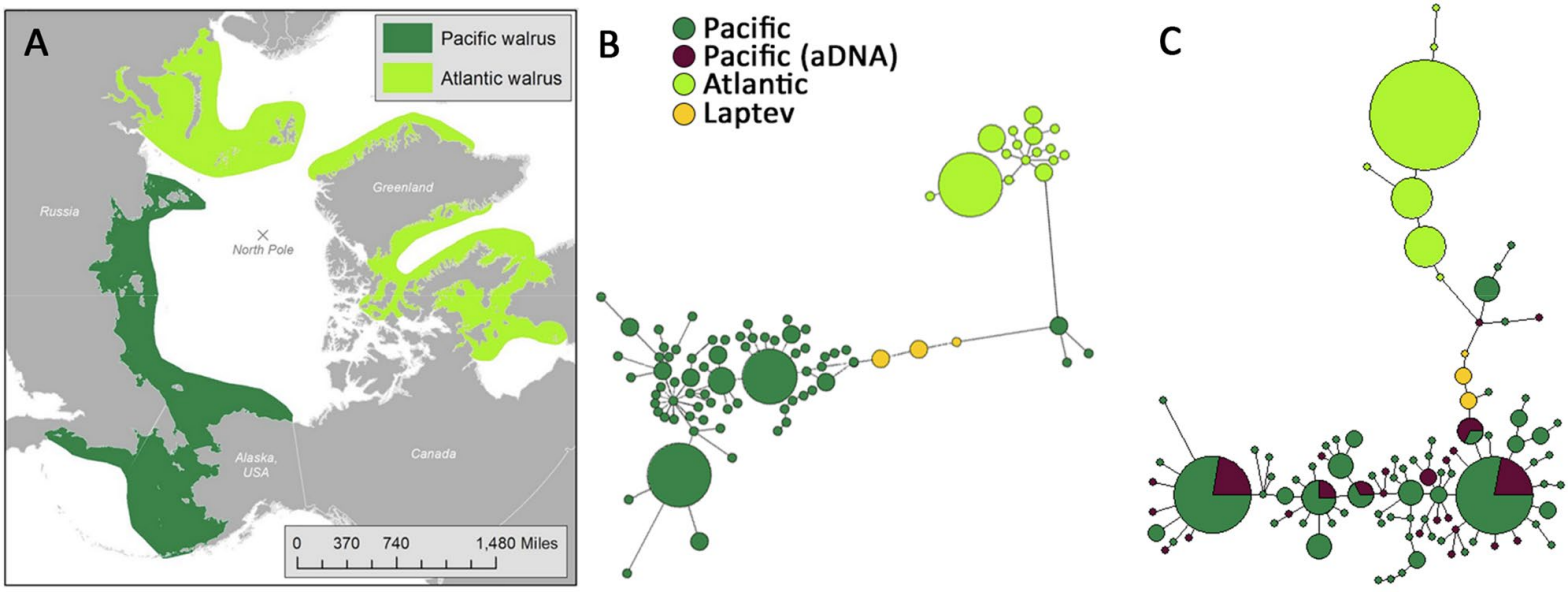
Haplotype networks for the three walrus subspecies. (**A**) Map depicting the modern ranges of *O. r. rosmarus divergens* and *O. r. rosmarus*. (**B**) Median-joining network based on the longest possible alignment (644 bp, n = 123). Node radii are proportional to the number of haplotypes, and branch lengths are proportional to the number of nucleotide differences separating two haplotypes. (**C**) Median-joining network based on a shorter alignment that also includes aDNA samples (196 bp, n = 149). Entirely purple nodes represent ancient haplotypes that are extinct or unobserved among modern samples.

**Figure 3:**
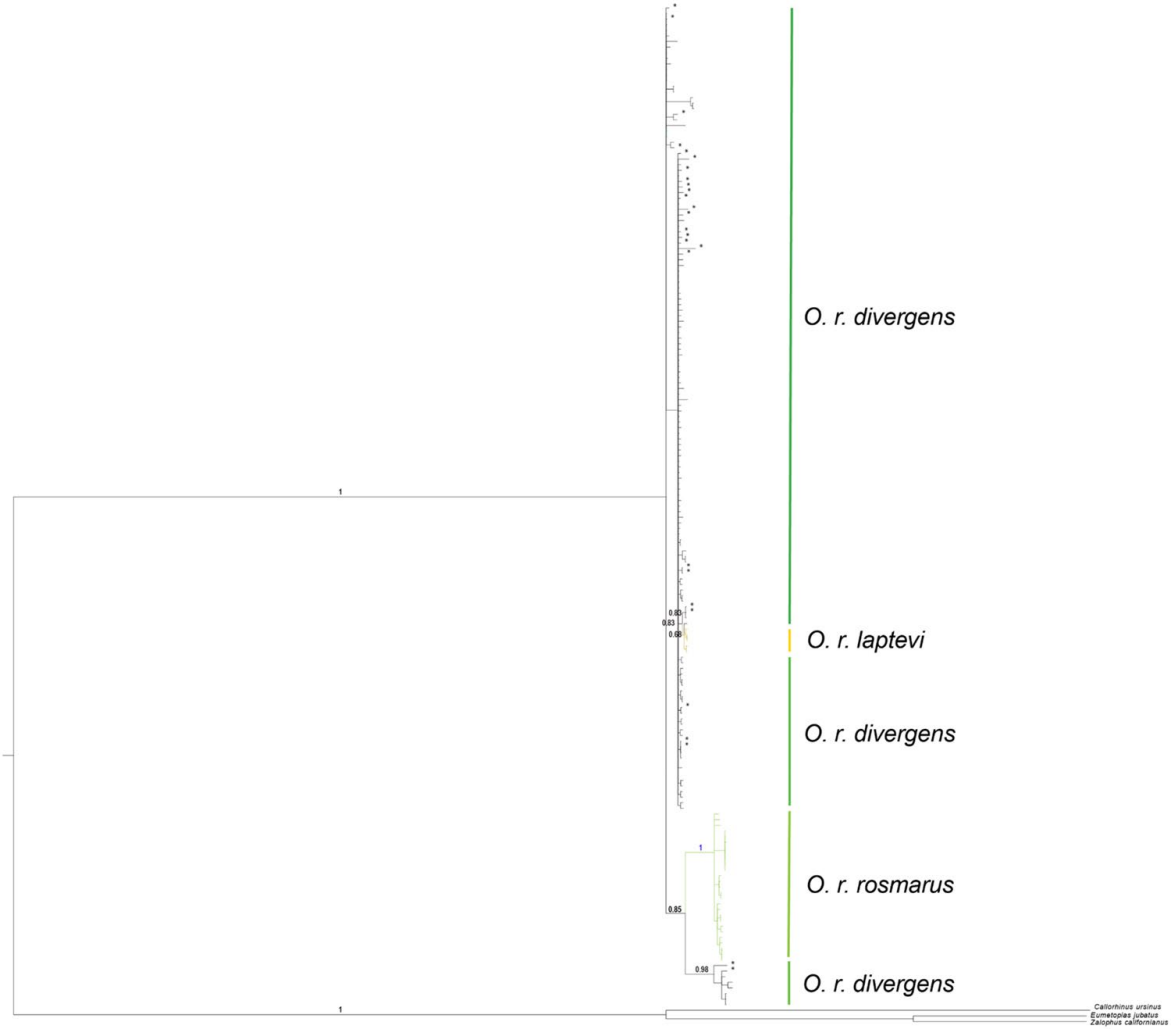
Bayesian phylogenetic tree estimated from the short alignment (196 bp of mitochondrial control region). Pacific Walruses (*Odobenus rosmarus divergens*) are shown in black, Atlantic Walruses (*O. r. rosmarus*) in green, and Laptev Walruses (*O. r. laptevi*) in orange. Asterisks indicate archaeological Pacific Walrus specimens.

The 26 aDNA sequences included 23 distinct haplotypes, 17 of which were unobserved in the modern samples. These 17 sequences represent either extinct haplotypes or haplotypes that were unsampled in the modern population by chance. Ancient haplotypes did not form obvious clusters or clades in either the haplotype networks (Fig. 2) or the phylogenetic tree (Fig. 3) and were evenly distributed throughout modern samples. In addition, no obvious structuring of modern Pacific Walruses is evident in either the haplotype networks or the phylogenetic tree. The Laptev subspecies did not form a clade and is therefore not a phylogenetically supported subspecies, appearing instead to represent a geographically isolated population of Pacific Walrus.

**Figure 4:**
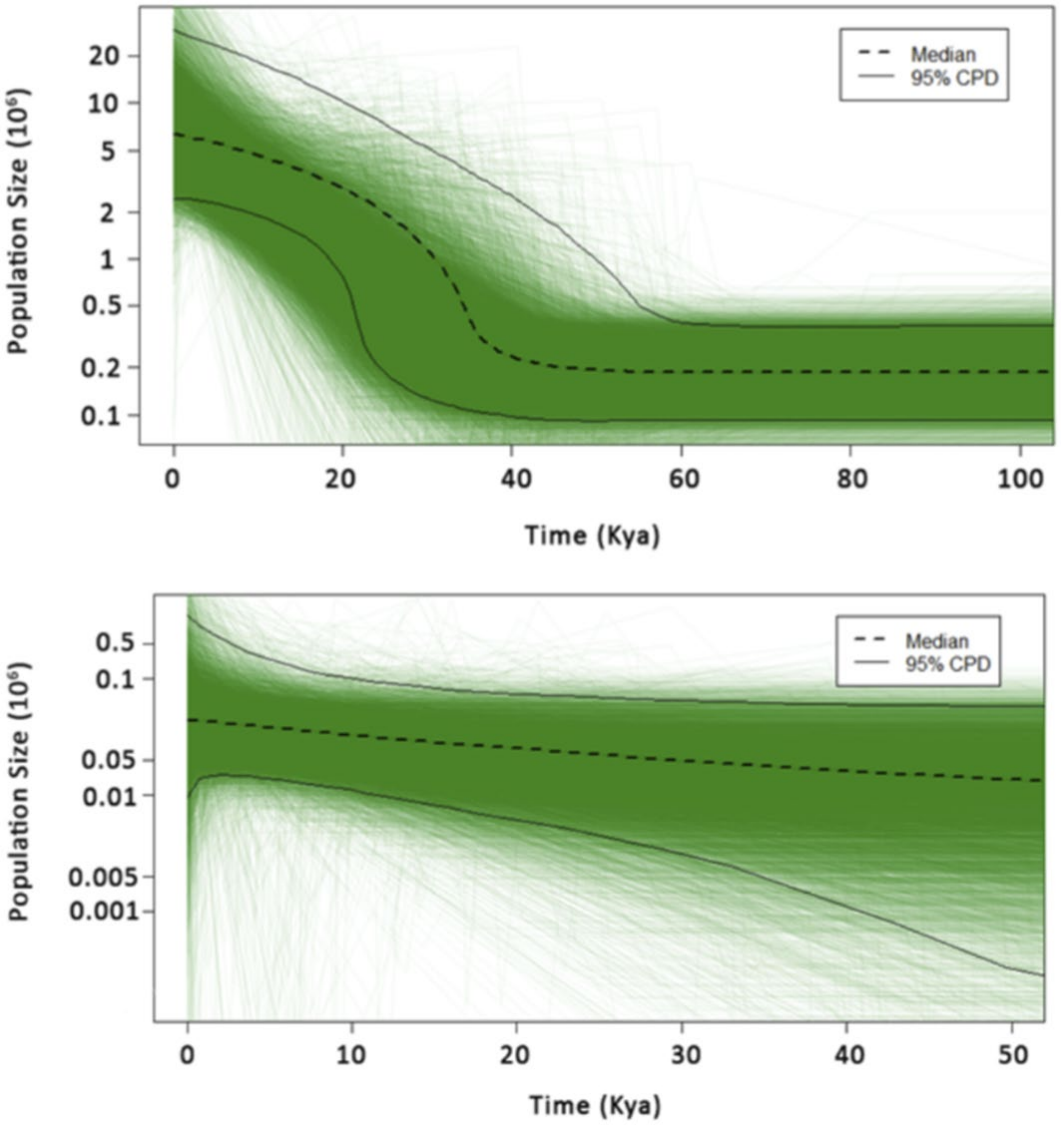
Extended Bayesian Skyline Plot of Pacific Walruses (top) and Atlantic Walruses (bottom) based on sequences from the long (644 bp) alignment. Green lines represent individual population trajectories in the posterior distribution. 95% of the coalescent events occurred in the last 100 Kya for Pacific Walruses and 50 Kya for Atlantic Walruses.

The Extended Bayesian Skyline Plots concur with the negative Fu’s* F*_*s*_ values for both subspecies, with a dramatic population size increase detected in Pacific Walruses and a relatively minor increase in Atlantic walruses (Fig. 4 ). The sudden growth of Pacific Walruses began approximately 40 Kya, and the sequence data inform the effective population size only to 100 Kya (Fig. S2). For Atlantic Walruses, the data were informative to 50 Kya (Fig. S3).

## Discussion

Pacific Walruses are thought to have numbered over 200,000 individuals prior to the onset of commercial hunting in the 19th century^17^. Over the span of only about 200 years, this number was reduced to approximately 50,000–100,000^5,6^. Despite this, our results indicate genetic diversity remained relatively unchanged over this period. The aDNA fragment we recovered was only 196 bp in length, but because it spanned part of the hypervariable mitochondrial control region, the alignment contained a high number of variable sites and distinct haplotypes (Table 2). Both nucleotide and haplotype diversity declined slightly between ancient and modern samples, but this change is negligible when compared to that of extant Atlantic Walruses. Similar results have been obtained for several other marine mammal species that underwent rapid and dramatic bottlenecking as a result of uncontrolled industrial hunting, particularly those that—like Pacific Walruses—had historically large population sizes and little genetic substructure. Our results are also consistent with the findings of^7^, who found that genetic signatures of bottlenecks were stronger in pinniped species that breed on land than species that breed on ice. These authors suggested that this is because terrestrially breeding pinnipeds were more accessible to hunters and thus suffered a higher proportional loss due to overhunting. This study provides additional evidence that highly vagile and panmictic marine mammals may be especially resistant to loss of genetic variation and inbreeding depression as habitat loss and human exploitation reduce their numbers. While obviously not impervious to extinction, such species may be less susceptible to the compounding effects of the extinction vortex—meaning habitat preservation should be the primary focus of management programs. Future habitat fragmentation that results in population substructure is likely to accelerate genetic erosion in Pacific Walruses and negate the protection conferred by their present panmixia. Furthermore, that Pacific Walruses breed on ice may have provided some refuge from commercial hunting, but it is now likely a vulnerability because climate change is reducing the extent of summer sea ice.

Although many potentially extinct haplotypes were detected, suggesting a temporal shift in genetic composition during the overhunting period, these haplotypes did not form clades or exhibit any obvious clustering in the haplotype network (Fig. 2) or phylogenetic tree (Fig. 3). This is consistent with this species being virtually panmictic, meaning even highly spatially concentrated hunting efforts would have been unlikely to wipe out entire genetic lineages. In contrast, two lineages of Atlantic Walruses were completely extirpated from the Canadian Maritimes region during the same period^55^. This Maritimes subpopulation was genetically and morphologically distinct from other Atlantic Walruses and inhabited the southernmost edge of the subspecies’ range. That there were no shared haplotypes between the extinct Maritimes and modern Atlantic Walruses suggests that the former may have been locally adapted to the warmer southern climate; regardless, the variation previously harbored by the Maritimes subpopulation has been lost. The highly divergent responses of the closely related Pacific and Atlantic subspecies likely reflects their distinct population structures. Unlike the panmictic Pacific Walruses, Atlantic Walruses are composed of an estimated eight distinct breeding stocks^56,57^. Other marine mammal species that exhibited strong population structure prior to the overhunting period—such as Hawaiian Monk Seals^58^, Galapagos Fur Seals^59^, and Sea Otters^27^—have low levels of genetic variation today and have failed to recover to pre-exploitation levels.

The Extended Bayesian Skyline Plot analysis, which was based on the longer 644 bp alignment, indicates Pacific Walruses began a substantial population expansion approximately 40 Kya (Fig. 4), which represents the middle of the Wisconsin Glacial Episode. During this period, the Cordilleran and Laurentide ice sheets expanded into much of the modern walrus’s range, probably providing extensive favorable habitat for the species. Although this analysis was less informative for Atlantic Walruses, fossil remains in the Canadian Maritimes suggest this subspecies also reached its greatest southern extent during the Wisconsin Glacial Episode^55^. Together, these results suggest walruses thrived when the planet was cooler and ice more widespread. Although it is well established that walruses are highly dependent on summer ice for survival, this provides yet more evidence that the species is highly specialized to the Arctic and that global warming has the potential to eradicate suitable walrus habitat completely. A recent population model estimated that the population size of Pacific Walruses in 2015 was 42% of what it was in 1975, and because walrus harvests are largely restricted to subsistence hunting now, the decline is likely due to summer sea ice loss over recent decades^60^.

Consistent with previous studies, both our short and long alignments indicate Pacific Walruses harbor considerably more genetic variation than Atlantic Walruses^23,33,56^. This could be because Atlantic Walruses derive from a relatively recent founding event from Pacific Walruses, or because the bottleneck they experienced during overhunting was much more intense (or both). Our phylogenetic analysis suggests Atlantic Walruses represent a recent colonization from the Pacific to the Atlantic Ocean (Fig. 3). This contrasts with previous suggestions that modern walruses originated in the Atlantic Ocean, colonized the Pacific Ocean in the Pleistocene, and then experienced population loss in the Atlantic^61,62^, but is consistent with fossil evidence indicating a Pacific origin^63,64^. A larger, multi-locus dataset is needed to answer this question more definitely and to date colonization events, and the results would provide a highly informative story about how genetic diversity is shaped by population expansions, founding events, and bottlenecks. If Atlantic Walruses derive from a single founding event from the Pacific, it would imply this event caused a substantially larger reduction in genetic diversity than the widespread intensive hunting of the last few centuries. Regardless of its cause, the low variation present in Atlantic Walruses, combined with their higher degree of population structure, means they are probably much more susceptible to entering the extinction vortex than Pacific Walruses.

Our results indicate that the Laptev Walrus is not a phylogenetically supported subspecies of *O. rosmarus*. Consistent with the findings of^33^, the Laptev population is clearly nested within the Pacific Walrus clade and would be more accurately classified as the westernmost population of the Pacific Walrus. However, there were no shared haplotypes between the two, they are separated by a substantial geographic distance, and there is no known gene flow between them. It is therefore unlikely that recolonization of the Laptev Sea would occur should the current population become locally extirpated. Because walruses are highly influential components of their marine ecosystems, the Laptev population should still be considered an important and isolated peripheral population for management purposes. Furthermore, it is unknown when or why the Laptev population became isolated from the rest of the Pacific species. It may be that the period of intensive overhunting fragmented the species’ range so effectively that it eliminated gene flow between previously connected localities. Alternatively, the Laptev population may have become isolated prior to this period due to some unknown biogeographic phenomenon. A larger, multi-locus dataset and a greater sample size of Laptev Walruses are needed to date when they became endemic to the Laptev Sea and whether their genomes show evidence of adaptation to the Laptev Sea specifically, which should provide clues as to the cause of their present isolated  distribution. This is highly relevant to their conservation, as management plans typically aim to rejoin populations that were fragmented abruptly by human exploitation (such as by facilitated gene flow), whereas populations that were separated by natural vicariance events are better served by managing them as distinct stocks^65^.

This study made use of ancient DNA to directly assess the impact of intensive commercial hunting on genetic variation in Pacific Walruses, but because nuclear DNA can only be easily obtained in modern samples, some questions about the status of this species remain unanswered. Future studies using large, multi-locus datasets could be used to estimate gene flow among the three subpopulations to better assess their degree of isolation, as well as estimate when the Atlantic and Laptev Walrus lineages dispersed from the Pacific Ocean. In addition, genomic-level analyses can measure the degree of inbreeding depression and mutational load by measuring runs of homozygosity (ROH) and the proportion of loss-of-function SNPs^66^, which are more direct measures of fitness than nucleotide and haplotype diversities and thus are more useful for assessing conservation status. The availability of a scaffold-level genome assembly for *O. rosmarus*, published in 2015, further enhances the utility of multi-locus datasets from this species^67^.

### Supplementary Information

Supplementary Information.

## Data Availability

The sequences generated for this project are available on GenBank (OQ183334–OQ183410) and a link is also provided through National Science Foundation’s Arctic Data Center.
