# Supplementary material for: Ancient DNA indicates a century of overhunting did not reduce genetic diversity in Pacific Walruses (Odobenus rosmarus divergens)
Source: Sci Rep. 2024 Apr 8;14:8257. doi: 10.1038/s41598-024-57414-2 (PMC11001934; doi:10.1038/s41598-024-57414-2)
Supplement: Supplementary file 1 — Supplementary Information. [file 41598_2024_57414_MOESM1_ESM.docx]

**Ancient DNA indicates a century of overhunting did not reduce genetic diversity in Pacific Walruses (*Odobenus rosmarus divergens*)**

Kendall K. Mills*^1,2^, Kyndall P.B. Hildebrandt^1^, Kathryn M. Everson^1,3^, Lara Horstmann^4^, Nicole Misarti^5^, and Link E. Olson^1^

^1^ Department of Mammalogy, University of Alaska Museum, 1962 Yukon Drive, Fairbanks, Alaska 99775, USA

^2^ Department of Biology and Wildlife, University of Alaska Fairbanks, Fairbanks AK 99775, USA

^3^Department of Integrative Biology, Oregon State University, 2701 SW Campus Way, Corvallis, OR 97331, USA

^4^ College of Fisheries and Ocean Sciences, University of Alaska Fairbanks, Fairbanks AK 99775, USA

^5^ Water and Environmental Research Center, University of Alaska Fairbanks, Fairbanks AK 99775, USA

*Corresponding author: Kendall K. Mills

# Supplementary Materials

**
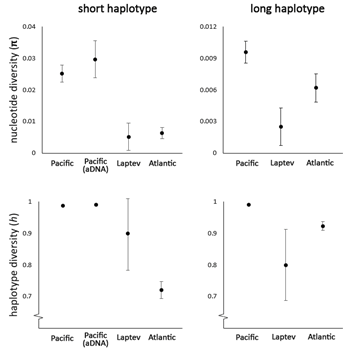
**

**Figure S1**. Nucleotide and haplotype diversity estimated from the short (196 bp) and long (644 bp) alignments for each subspecies. The short alignment includes 16 archeological Pacific Walrus samples, whereas the long alignment contains only modern samples. Error bars represent 95% confidence intervals. Estimates were calculated for each sample set in R using PEGAS (Paradis 2010).


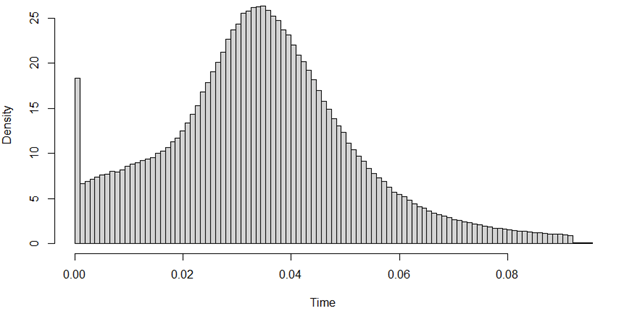


**Figure S2**. Histogram of tree event times (in logarithmic scale) in the Extended Bayesian Skyline analysis for Pacific walruses, depicting 95% of all tree events.


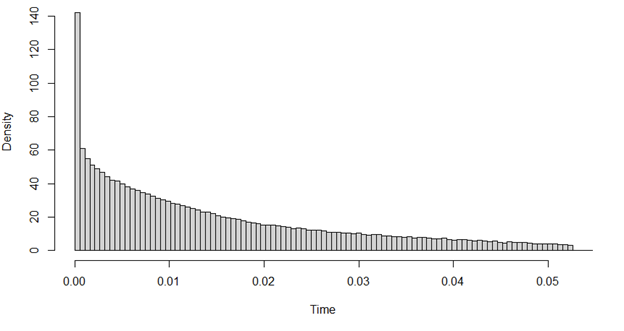


**Figure S3**. Histogram of tree event times (in logarithmic scale) in the Extended Bayesian Skyline analysis for Atlantic walruses, depicting 95% of all tree events.

**Table S1**. List of specimens used in this study. In most cases, identifier is the globally unique identifier (GUID) for specimens archived in the University of Alaska Museum’s Mammal collection (UAM:Mamm) or Archaeology collection (UAM:Arc). Conventional catalog numbers for specimens archived in the Mammal collection of the U.S. National Museum of Natural History, Smithsonian Institution (USNM). Parenthetical alphanumeric identifiers refer to those samples also used in Clark et al. (2019). All samples derive from Alaska, USA. Sex is female (F), male (M), or unknown (U).

| **Identifier** | **Repository** | **Year** | **Sex** | **Tissue** | **Locality** |
| --- | --- | --- | --- | --- | --- |
| UAM:Mamm:16588 | UAM | 1933 | F | Bone (cranium) | St. Lawrence |
| UAM:Mamm:11691 | UAM | 1957 | F | Bone (cranium) | St. Lawrence |
| UAM:Mamm:5041 | UAM | 1961 | M | Bone (tibia) | Point Hope |
| UAM:Mamm:5049 | UAM | 1961 | F | Bone (humerus) | Point Hope |
| UAM:Mamm:4816 | UAM | 1961 | U | Bone (scapula) | Elephant Point |
| UAM:Mamm:146252 | UAM | 1965 | U | Bone (cranium) | Wainwright |
| UAM:Mamm:12084 | UAM | 1978 | M | Bone (cranium) | Round Island/Nushagak Bay |
| UAM:Mamm:12082 | UAM | 1978 | M | Bone (cranium) | Nushagak Bay |
| UAM:Mamm:12077 | UAM | 1978 | M | Bone (mandible) | Round Island |
| UAM:Mamm:12080 | UAM | 1978 | M | Bone (cranium) | Nushagak Bay |
| UAM:Mamm:146251 | UAM | 1981 | U | Bone (scapula) | Cape Pierce |
| UAM:Mamm:53995 | UAM | 1991 | F | Muscle | southeast of Nunivak Island |
| UAM:Mamm:54207 | UAM | 1991 | U | Muscle | south of Cape Navain to Cape Olyutorsky |
| UAM:Mamm:54245 | UAM | 1991 | U | Muscle | southeast of St. Lawrence Island |
| UAM:Mamm:54042 | UAM | 1991 | U | Muscle | south of Cape Navain to Cape Olyutorsky |
| UAM:Mamm:54060 | UAM | 1991 | U | Muscle | south of Cape Navain to Cape Olyutorsky |
| UAM:Mamm:54041 | UAM | 1991 | U | Muscle | south of Cape Navain to Cape Olyutorsky |
| UAM:Mamm:54325 | UAM | 1991 | U | Muscle | southeast of St. Lawrence Island |
| UAM:Mamm:54299 | UAM | 1991 | M | Muscle | southeast of St. Lawrence Island |
| UAM:Mamm:54247 | UAM | 1991 | F | Muscle | southeast of Nunivak Island |
| UAM:Mamm:54177 | UAM | 1991 | U | Muscle | southeast of Nunivak Island |
| UAM:Mamm:54258 | UAM | 1991 | U | Muscle | south of Cape Navain to Cape Olyutorsky |
| UAM:Mamm:54055 | UAM | 1991 | U | Muscle | south of Cape Navain to Cape Olyutorsky |
| UAM:Mamm:54039 | UAM | 1991 | U | Muscle | south of Cape Navain to Cape Olyutorsky |
| UAM:Mamm:54322 | UAM | 1991 | U | Muscle | southeast of St. Lawrence Island |
| UAM:Mamm:54008 | UAM | 1991 | F | Muscle | southeast of Nunivak Island |
| UAM:Mamm:53952 | UAM | 1991 | U | Muscle | southeast of Nunivak Island |
| UAM:Mamm:54145 | UAM | 1991 | U | Muscle | southeast of Nunivak Island |
| UAM:Mamm:54255 | UAM | 1991 | U | Muscle | south of Cape Navain to Cape Olyutorsky |
| UAM:Mamm:54061 | UAM | 1991 | U | Muscle | south of Cape Navain to Cape Olyutorsky |
| UAM:Mamm:54252 | UAM | 1991 | U | Muscle | south of Cape Navain to Cape Olyutorsky |
| UAM:Mamm:54286 | UAM | 1991 | U | Muscle | southeast of St. Lawrence Island |
| UAM:Mamm:54257 | UAM | 1991 | U | Muscle | south of Cape Navain to Cape Olyutorsky |
| UAM:Mamm:54323 | UAM | 1991 | U | Muscle | southeast of St. Lawrence Island |
| UAM:Mamm:54357 | UAM | 1991 | U | Muscle | southeast of St. Lawrence Island |
| UAM:Mamm:54216 | UAM | 1991 | U | Muscle | southeast of St. Lawrence Island |
| UAM:Mamm:54244 | UAM | 1991 | U | Muscle | southeast of St. Lawrence Island |
| UAM:Mamm:54285 | UAM | 1991 | U | Muscle | southeast of St. Lawrence Island |
| UAM:Mamm:53963 | UAM | 1991 | F | Muscle | southeast of Nunivak Island |
| UAM:Mamm:54254 | UAM | 1991 | M | Muscle | south of Cape Navain to Cape Olyutorsky |
| UAM:Mamm:54316 | UAM | 1991 | U | Muscle | southeast of St. Lawrence Island |
| UAM:Mamm:54397 | UAM | 1991 | U | Muscle | southeast of Nunivak Island |
| UAM:Mamm:54260 | UAM | 1991 | U | Muscle | south of Cape Navain to Cape Olyutorsky |
| UAM:Mamm:54054 | UAM | 1991 | M | Muscle | south of Cape Navain to Cape Olyutorsky |
| UAM:Mamm:54324 | UAM | 1991 | U | Muscle | southeast of St. Lawrence Island |
| UAM:Mamm:54220 | UAM | 1991 | U | Muscle | southeast of St. Lawrence Island |
| UAM:Mamm:54346 | UAM | 1991 | F | Muscle | southeast of St. Lawrence Island |
| UAM:Mamm:54281 | UAM | 1991 | U | Muscle | southeast of St. Lawrence Island |
| UAM:Mamm:54284 | UAM | 1991 | U | Muscle | southeast of St. Lawrence Island |
| UAM:Mamm:109526 | UAM | 1992 | F | Bone (tooth) | Diomede |
| UAM:Mamm:109577 | UAM | 1992 | F | Bone (tooth) | Diomede |
| UAM:Mamm:60352 | UAM | 2001 | F | Muscle | 31-40 miles from Gambell |
| UAM:Mamm:60354 | UAM | 2001 | M | Muscle | 31-40 miles from Gambell |
| UAM:Mamm:60350 | UAM | 2001 | F | Muscle | 11-20 miles from Gambell |
| UAM:Mamm:60353 | UAM | 2001 | M | Muscle | 31-40 miles from Gambell |
| UAM:Mamm:60383 | UAM | 2001 | M | Muscle | 11-20 miles from Gambell |
| UAM:Mamm:60346 | UAM | 2001 | F | Muscle | 11-20 miles from Gambell |
| UAM:Mamm:60210 | UAM | 2001 | M | Muscle | 6 miles SW of south end of Little Diomede Island |
| UAM:Mamm:88499 | UAM | 2002 | F | Muscle | 11-20 miles from Gambell |
| UAM:Mamm:101094 | UAM | 2002 | F | Kidney | 8 mi NE of Savoonga |
| UAM:Mamm:99969 | UAM | 2003 | M | Muscle | 30 miles northeast of Savoonga |
| UAM:Mamm:99961 | UAM | 2003 | F | Muscle | 35 mile northwest of Savoonga |
| UAM:Mamm:47754 | UAM | 2003 | M | Muscle | 17 miles northeast of Gambell |
| UAM:Mamm:97832 | UAM | 2004 | M | Muscle | Diomede |
| UAM:Mamm:128832 | UAM | 2009 | F | Soft Tissue | Savoonga |
| UAM:Mamm:128005 | UAM | 2009 | F | Soft Tissue | Gambell |
| S090052 | USFWS | 2009 | F | Soft Tissue | Savoonga |
| S090021 | USFWS | 2009 | F | Soft Tissue | Savoonga |
| UAM:Mamm:128004 | UAM | 2009 | F | Soft Tissue | Gambell |
| UAM:Mamm:128883 | UAM | 2009 | M | Soft Tissue | Savoonga |
| UAM:Mamm:116517 | UAM | 2012 | M | Muscle | Gambell |
| UAM:Mamm:116559 | UAM | 2012 | F | Muscle | Gambell |
| UAM:Mamm:116581 | UAM | 2012 | F | Kidney | Gambell |
| UAM:Mamm:125304 | UAM | 2014 | M | Liver | Savoonga |
| UAM:Mamm:125305 | UAM | 2014 | M | Liver | Savoonga |
| UAM:Mamm:125309 | UAM | 2014 | U | Liver | Savoonga |
| UAM:Mamm:125310 | UAM | 2014 | M | Liver | Savoonga |
| UAM:Mamm:125316 | UAM | 2014 | M | Liver | Savoonga |
| UAM:Mamm:125318 | UAM | 2014 | M | Liver | Savoonga |
| UAM:Mamm:125289 | UAM | 2014 | F | Bone (tooth) | Gambell |
| UAM:Mamm:133127 | UAM | 2015 | U | Skin* | Cape Lisburne |
| UAM:Mamm:133128 | UAM | 2015 | U | Skin* | Cape Lisburne |
| UAM:Mamm:133129 | UAM | 2015 | U | Skin* | Cape Lisburne |
| UAM:Mamm:133130 | UAM | 2015 | U | Skin* | Cape Lisburne |
| UAM:Mamm:133131 | UAM | 2015 | U | Skin* | Cape Lisburne |
| UAM:Mamm:133134 | UAM | 2015 | U | Skin* | Cape Lisburne |
| UAM:Mamm:133135 | UAM | 2015 | U | Skin* | Cape Lisburne |
| UAM:Mamm:133137 | UAM | 2015 | U | Skin* | Cape Lisburne |
| UAM:Mamm:133139 | UAM | 2015 | U | Skin* | Cape Lisburne |
| UAM:Arc:UA75-009 | UAM | Arc. | U | Bone (cranial fragment) | Old Tigara |
| UAM:Arc:UA75-009 | UAM | Arc. | U | Bone (bulla) | Old Tigara |
| SL1-676 | UIC | Arc. | U | Bone (mandible) | Pingusugruk |
| SL2-KQBPX | UIC | Arc. | U | Bone (cranium) | Pingusugruk |
| SL1-855 | UIC | Arc. | U | Bone (humerus) | Pingusugruk |
| SL2-HMFLI | UIC | Arc. | U | Bone (radius) | Pingusugruk |
| SL2-3178 | UIC | Arc. | U | Bone (scapula) | Pingusugruk |
| NOAT-00092-27386 | ARCC | Arc. | U | Bone (rib) | Maiyumerak Creek (XBM-131) |
| UAM:Arc:UA68-070-0453 | UAM | Arc. | M | Bone (baculum) | Kukulik |
| UAM:Arc:UA68-070-1186 | UAM | Arc. | U | Bone (cranium) | Kukulik |
| UAM:Arc:UA68-086-0004 | UAM | Arc. | U | Bone (mandible) | Kiyalighaq |
| UAM:Arc:UA68-086-0066 | UAM | Arc. | M | Bone (baculum) | Kiyalighaq |
| UAM:Arc:UA68-011-1028 | UAM | Arc. | U | Bone (cranium) | Old Togiak |
| UAM:Arc:UA65-011-2855 | UAM | Arc. | U | Bone (scapula) | Old Togiak |
| UAM:Arc:XPM-00001-3413 | UAM | Arc. | U | Bone (metatarsal) | Hot Springs |
| UAM:Arc:XPM-00001-3469 | UAM | Arc. | U | Bone (radius) | Hot Springs |
| UAM:Arc:XPM-00001-6790 | UAM | Arc. | U | Bone (sternebrae) | Hot Springs |
| UAM:Arc:XPM-00001-27006 | UAM | Arc. | U | Bone (humerus) | Hot Springs |
| UAM:Arc:XPM-00001-40221 | UAM | Arc. | U | Bone (metatarsal) | Hot Springs |
| UAM:Arc:UA2012-052-2564 | UAM | Arc. | U | Bone (mandible) | Nuwuk |
| UAM:Arc:UA2012-051-5338 | UAM | Arc. | U | Bone (mandible) | Carter Collection/Birnirk |
| UAM:Arc:UA2012-051-4538 | UAM | Arc. | U | Worked Bone | Carter Collection/Birnirk |
| UAM:Arc:UA2012-051-9274 | UAM | Arc. | U | Bone (cranium) | Carter Collection/Birnirk |
| UAM:Arc:UA2012-051-9194 | UAM | Arc. | U | Bone (cranial fragment) | Carter Collection/Birnirk |
| D92080 | USFWS | 1992 | U | Soft tissue | Diomede |

*sampled on site of mortality event; skin sample from same individual archived with this same catalog number

**References**

Clark, C. T., Horstmann, L., Vernal, A. de, Jensen, A. M. & Misarti, N. Pacific walrus diet across 4000 years of changing sea ice conditions. Quat. Res. 108, 26–42 (2022).

Paradis, E. pegas: an R package for population genetics with an integrated–modular approach. Bioinformatics 26, 419–420 (2010).
